# Screening and Identification of Protease-Producing Microorganisms in the Gut of *Gryllotalpa orientalis* (Orthoptera: Gryllotalpidae)

**DOI:** 10.3390/insects15080629

**Published:** 2024-08-21

**Authors:** Xiang Zheng, Lu Zhao, Fangtong Wu, He Zhou, Fuming Shi

**Affiliations:** 1 Laboratory of Enzyme Preparation, Hebei Research Institute of Microbiology Co., Ltd., Baoding 071051, China; 569186912@163.com (X.Z.); 15832297069@139.com (L.Z.); yayawu710@hotmail.com (F.W.); zhouhebio@163.com (H.Z.); 2 Institute of Life Science and Green Development, College of Life Science, Hebei University, Baoding 071002, China

**Keywords:** insect, gut microorganism, screening, identification, dDDH, ANI

## Abstract

**Simple Summary:**

This study employed microbial culturing to isolate four strains of bacteria with protease activity from the gut of an omnivorous insect. The four bacteria were precisely identified using whole-genome relatedness indices, based on conventional bacterial species identification methods. Furthermore, their whole-genome functional information was analyzed and compared, establishing a technical foundation for the development and utilization of protease-producing microorganisms from the insect gut.

**Abstract:**

The insect gut harbors a diverse array of functional microorganisms that warrant further exploration and utilization. However, there is currently a paucity of research reports on the discovery of protease-producing microorganisms with industrial application value in the gut. Here, we employed microbial culturing to screen and identify the protease-producing microorganisms in the gut extract of *Gryllotalpa orientalis*. Based on morphological, physiological, and biochemical characterization, 16S rRNA sequencing, as well as ANI and dDDH values of whole genome, the protease-producing strains isolated from the insect gut were identified as *Priestia aryahattai* DBM-1 and DX-4, *P. megaterium* DX-3, and *Serratia surfactantfaciens* DBM-5. According to whole-genome analysis, strain DBM-5, which exhibited the highest enzyme activity, possesses abundant membrane transport genes and carbohydrate metabolism enzymes. In contrast, strains DX-3 and DX-4 not only have the ability to hydrolyze proteins but also demonstrate the capability to hydrolyze plant materials. Furthermore, strains that are closely related tend to have similar metabolic product gene clusters in their genomes. The screening and identification of protease resources are essential for the subsequent development and utilization of gut functional microorganisms and genetic resources in insects.

## 1. Introduction

The gut of insects is rich in microbial resources, serving as a resource pool for exploring functional and active substances [1]. As more and more biological characteristics of gut microbiota were being uncovered, there was a growing exploration and utilization of potential functional microorganisms and their functional genes [2,3]. The provision of abundant nutrients by functional microorganisms to insects may play a critical role in the formation of mutually beneficial relationships in biological evolution [1]. This was achieved through the secretion of various necessary hydrolytic enzymes for nutrient decomposition, digestion, and absorption, ensuring a sufficient nutritional supply [4]. Microbial enzymes have been widely utilized across various industries [5,6] due to their environmental friendliness, potential catalytic ability, capacity to optimize production processes, and capability for in vitro expression through gene recombination and protein engineering [7]. The exploration of microbial enzyme resources will be a pivotal focus in modern industrial biotechnology [8].

Currently, there have been reports of enzyme-producing microorganisms in the gut of various insects, such as the gut of termites, which were abundant in resources of lignocellulose-degrading microorganisms [9]. The gut microbiota of *Tenebrio molitor* larvae demonstrate a superior ability in the biodegradation of cellulose, polyethylene, and polystyrene waste [10,11]. Several trypsin- and chymotrypsin-like proteases have been identified in *Periplaneta americana*, exhibiting high activity at alkaline pH levels, thus demonstrating significant potential for industrial applications [12,13]. However, there has been limited research on the screening and identification of gut microorganisms in Orthoptera. Most existing studies have focused on the structure and function of gut microbial communities in Orthoptera [14,15]. Meanwhile, microbial proteases have a wide range of applications including for the clean production of leather, flavor enhancement of food, washing aids, and silk degumming processes [16,17]. Therefore, isolating protease-producing microorganisms from the insect gut will enhance the available resources for protease application.

The Gryllotalpidae belongs to the taxon of Orthoptera and is considered one of the most distinctive groups within Orthoptera due to its unique appearance. *Gryllotalpa orientalis* are omnivorous soil-dwelling pests that primarily feed on the roots and stems of seedlings. Additionally, they also consume soil invertebrates such as insects and earthworms, indicating a high presence of animal and plant proteolytic enzymes in their gut [18]. This study utilized microbial culturing to screen protease-producing microorganisms from the gut of the *G. orientalis* [19]. The identified strains were characterized through morphological observation, physiological and biochemical characteristics, and 16S rRNA gene and whole-genome sequencing in order to explore enzymatic microbial resources from the insect gut [20].

## 2. Materials and Methods

### 2.1. Insects’ Collection and Dissection 

*G. orientalis* were collected from a farmland in Quyang County, Hebei Province, China. The body surface of the adult insects was disinfected with 75% alcohol, then rinsed thrice with sterile water, and then dissected under sterile conditions. The sterilized gut of each insect was then extracted and placed in 0.5 mL of PBS sterile buffer solution, with a pH of 7.2–7.4, containing one sterile steel ball. Under ice bath conditions, the gut samples were intermittently shaken until the gut tissue and contents were broken down, after which the steel balls were removed under sterile conditions [21,22]. The gut sample solution was centrifuged for 30 s at 1520× *g* at 4 °C. Subsequently, the supernatant was subjected to serial dilutions from 10^−1^ to 10^−6^ with PBS sterile buffer solution, resulting in a pH of 7.2–7.4. Following this, 100 μL of each diluted gut contents were evenly spread onto the corresponding protease-producing primary screening solid culture medium with the following composition (g/L): casein (10), peptone (2), yeast powder (1), NaCl (2), agar powder (15), pH 7.0–7.2 [23,24]. The gut bacteria were then cultured in this culture media at 37 °C for 48 h, with three replicates for each dilution. 

### 2.2. Screening of Proteolytic Bacteria

The strain which exhibited a hydrolysis circle around the colony in the protease primary screening medium indicated its capability to produce protease and hydrolyze casein. Such colonies were selected and repeatedly cultured at 37 °C in the secondary screening medium (skimmed milk powder 20 g/L, agar powder 20 g/L, pH 7.0–7.2) [25] until a single colony with a transparent ring appeared; then, the strain was preserved [26]. 

### 2.3. Morphological, Physiological, Biochemical, and Molecular Identification of Bacteria

The morphological observation of proteolytic bacteria was performed using Gram staining and a series of physiological and biochemical tests on the above bacteria, including the macromolecular material hydrolysis test (gelatin, starch, glycerol, etc.), sugar (alcohol) fermentation test, IMViC test [27] (indole test, hydrogen sulfide test, VP test, citrate test, etc. were conducted.

The genomic DNA of bacteria was extracted for molecular identification. The 16S rRNA gene sequence was amplified using universal primers 27F (5′-AGTTTGATCMTG GCTCAG-3′) and 1492R (5’-GGTTACCTTGTTACGACTT-3′) [28]. The amplified products were sequenced through commercial services provided by Sangon Biotech Co., Ltd. (Shanghai, China). The comparison of sequencing results was performed by BLAST through NCBI’s 16S ribosomal RNA sequences database (Bacteria and Archaea), using the Cluster W program of MEGA for multiple sequence alignment. Then, a phylogenetic tree was constructed by the neighbor-joining model of the MEGA 11.0 [29,30].

### 2.4. Whole-Genome Analysis of Proteolytic Bacteria

Bacterial cell suspensions in LB broth at the logarithmic growth phase were harvested. Genomic DNA was then extracted using magnetic beads, and the bacterial genomes was sequenced through commercial services provided by Sangon Biotech Co., Ltd. (Shanghai, China). Subsequently, genomes were constructed, and high-throughput sequencing was performed on the Illumina HiSeq sequencing platform [11]. The original sequencing data was evaluated and controlled through Fastp [31]. SPAdes [32] was utilized to assemble the sequencing data, and GapFiller [33] was employed to fill in gaps in the concatenated contig. Afterward, Pilon (https://github.com/broadinstitute/pilon, accessed on 22 July 2023) was used for sequence correction to complete genome splicing. Firstly, the evolutionary status of proteolytic bacteria was inferred based on the 16S rRNA gene sequence. Subsequently, OAT 0.93.1 (Orthologous Average Nucleotide Identity Tool, https://www.ezbiocloud.net/tools/orthoani, accessed on 2 August 2023) software was used to analyze the characteristics of ANI (Average nucleotide identity) value across the entire genome of strains [34], and genome-to-genome distance calculator 2.1 (https://ggdc.dsmz.de/, accessed on 6 September 2023) was used to calculate the dDDH (Digital DNA–DNA hybridization) value [35], in order to determine the taxonomic relationship of the proteolytic strains based on their genome sequence. NCBI-PGAP conducts genome analysis and predicts gene elements, including CDS, tRNA, and rRNA. The gene sequences were compared with multiple databases such as NR (http://ncbi.nlm.nih.gov/, accessed on 23 July 2023), COG (https://www.ncbi.nlm.nih.gov/COG/, accessed on 27 July 2023), PFAM (http://pfam.xfam.org/, accessed on 27 July 2023), GO (https://geneontology.org/, accessed on 29 July 2023), KEGG (http://www.kegg.jp, accessed on 29 July 2023), CAZy (http://www.cazy.org/, accessed on 27 July 2023), and CARD (https://card.mcmaster.ca/, accessed on 27 July 2023) using blastp with an evaluation threshold of ≤1 × 10^−5^ to obtain gene functional annotation information. Differential analysis of functional gene clusters among four proteolytic strains was performed using antiSMASH (https://antismash.secondarymetabolites.org/, accessed on 15 August 2023). Based on the Expasy database (https://enzyme.expasy.org/index.html, accessed on 13 August 2024), information on various types of proteases was retrieved. Then, the protease genes and their corresponding species were selected from the annotation results of strains in the KEGG ENZYME database based on this protease information. 

## 3. Results

### 3.1. Screening and Identification of Proteolytic Bacteria

**Figure 1:**
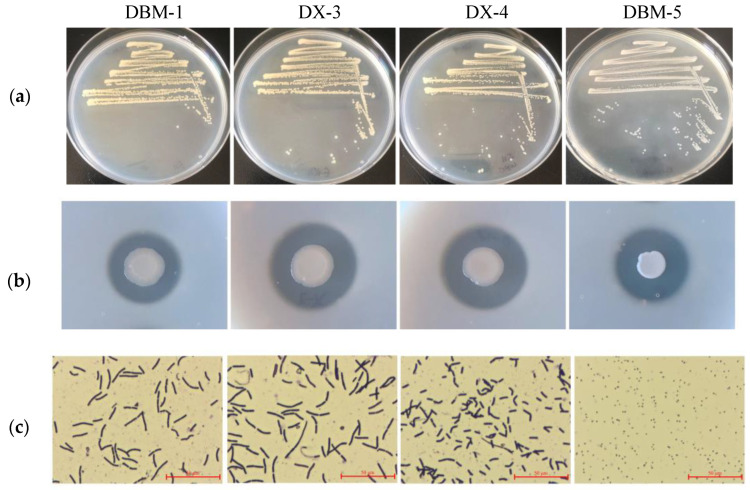
Screening of proteolytic bacteria in the gut of *G. orientalis*. (**a**) Screening with primary screening medium; (**b**) Characterization of protease production characteristics of strains using secondary screening medium; (**c**) Microscopic observation of the strain.

A total of 15 proteolytic bacteria were isolated from the gut extracts of *G. orientalis*. Among these, four strains exhibited higher protease activity and were designated as DBM-1, DX-3, DX-4, and DBM-5. According to the primary screening medium (Figure 1a), the colony morphology of strains DBM-1, DX-3, and DX-4 was similar—milky white, opaque, circular, with wrinkles on the edges. Strain DBM-5 was milky white and semitransparent with a smooth surface and raised center. Hydrolysis of the culture medium was evident around the DBM-5 colony, and red appeared around the colony as the cultivation time prolonged (Figure S1). The ratio of the transparent circle to the diameter of the colony in the secondary screening medium represents the ability of the strain to produce protease (Figure 1b). The strain which exhibited the most substantial enzyme production ability was DBM-5, followed by strains DX-3 and DX-4, and the strain with the weakest enzyme production ability was strain DBM-1. In microscopic morphology (Figure 1c), the strain DBM-5 appeared relatively smaller in comparison to the other three strains.

The physiological and biochemical analysis of proteolytic bacteria (Tables S1 and S2) revealed a strong ability to utilize carbohydrates in strains DBM-1, DX-3, and DX-4. In contrast to strains DBM-1 and DX-4, strain DX-3 exhibited limited hydrolysis capability in glycerol, D-lactose, D-galactose, and D-Toulon sugar. However, it demonstrated a unique ability to hydrolyze D-melezitose. Strain DX-4 demonstrated a higher capacity for hydrolyzing L-rhamnose, inositol, and α- methyl-D glucoside. In contrast, strain DBM-5 exhibited the ability to ferment various sugars and alcohols, produce acid, and secrete some hydrolytic enzymes. 

**Figure 2:**
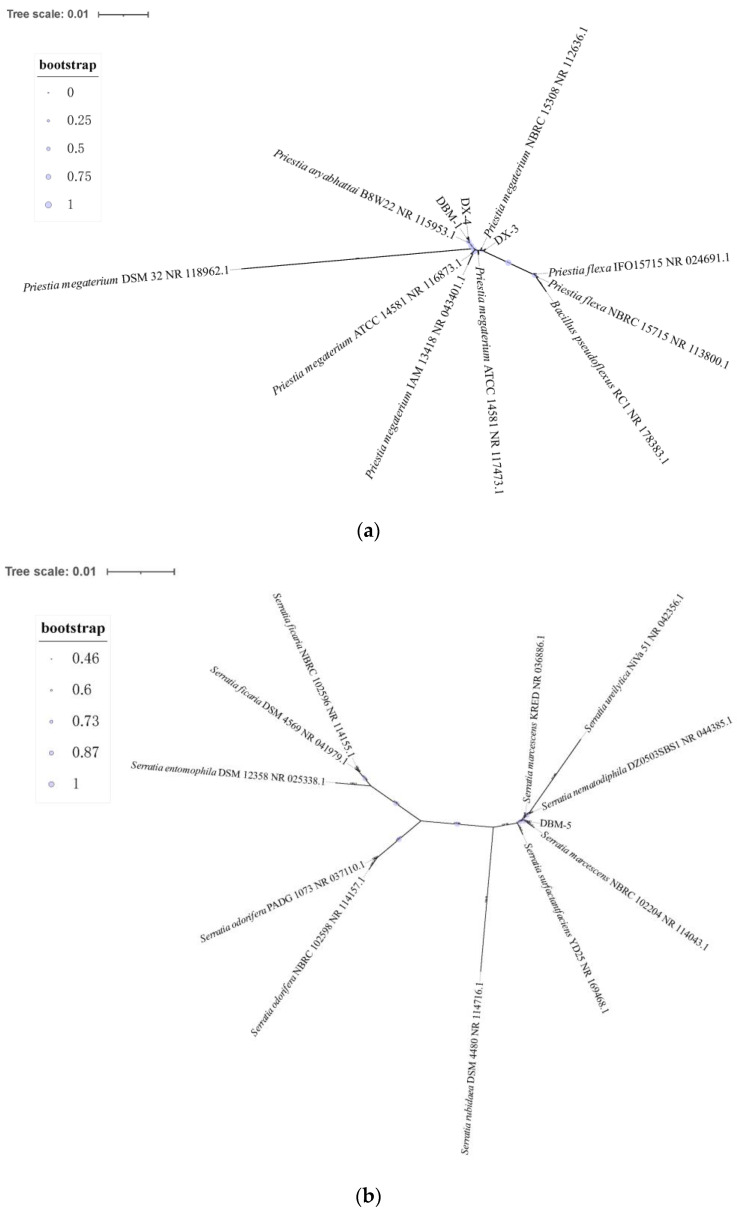
Unrooted evolutionary tree based on neighbor-joining of 16S rRNA gene of the strains in the gut of *G. orientalis*. (**a**) Unrooted evolutionary tree of strains DBM-1, DX-3, and DX-4; (**b**) Unrooted evolutionary tree of strain DBM-5.

Multiple sequence alignment was conducted for homologous sequences of four proteolytic strains, and unrooted evolutionary trees based on the 16S rRNA gene were constructed (Figure 2a,b). The closest relative to strains DBM-1 and DX-4 was *Priestia aryahattai* B8W22, while strain DX-3 showed the closest evolutionary distance to *P. megaterium* NBRC 15308, and strain DBM-5 had the closest evolutionary distance to *Serratia marcescens* NBRC 102204. Based on the morphology, physiological and biochemical experiments, 16S rDNA sequence alignment, and phylogenetic analysis, it was speculated that strains DBM-1 and DX-4 were classified as *P. aryabhattai*, strain DX-3 as *P. megaterium*, and strain DBM-5 as *S. marcescens*. 

### 3.2. Analysis of Proteolytic Bacteria through Whole-Genome Sequencing

**Figure 3:**
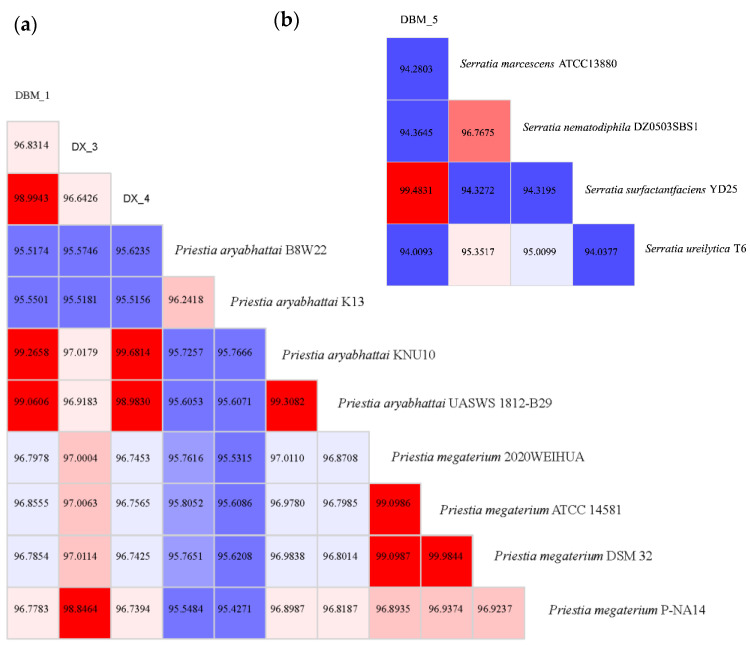
The ANI values of genome comparison between proteolytic bacteria and similar strains. (**a**) Genome ANI values of strains DBM-1, DX-3, and DX-4; (**b**) Genome ANI values of strain DBM-5. Notes: Different colors correspond to different levels of ANI values, with red indicated high ANI values and blue indicated low ANI values.

Based on the identification results of 16S rRNA gene sequencing, strains with close genetic relationships were screened for genomic ANI and dDDH values (Table 1 and Figure 3). The thresholds for ANI and dDDH were set at 95–96% and 70%, respectively, in accordance with the criteria for species differentiation [36]. The genome of strains DBM-1 and DX-4 were found to be most similar to the reference strain of *P. aryabhattai* UASWS 1812-B29, with ANI values of over 98% and dDDH values of over 91%. Although the ANI and dDDH values shared by strains DBM-1 and DX-4 with *P. megaterium* also exceed the same threshold, their values with *P. aryabhattai* were significantly higher than those of *P. megaterium*. Therefore, it was concluded that strains DBM-1 and DX-4 belong to the species *P. aryabhattai*. The genome of strain DX-3 was found to be most similar to *P. megaterium* P-NA14, leading to the conclusion that strain DX-3 belongs to the species *P. megaterium*. The genome identification results of strains DBM-1, DX-3, and DX-4 were consistent with the 16S rRNA gene sequence identification. According to the 16S rRNA gene identification, it was determined that there is a close genetic relationship between strains DBM-5 and *S. marcescens*. However, in whole-genome identification, it was observed that the ANI and dDDH values of strain DBM-5 and *S. marcescens* did not reach the same threshold, instead they shared 99.48% of the ANI value and 96.3% of the dDDH value with *S. surfactantfaciens* YD25. Therefore, DBM-5 was concluded to be *S. surfactantfaciens*.

The strain DX-4 possessed the largest genome, with a total length of 5,135,523 bp and the highest predicted number of coding genes (CDS) at 5222. However, it also had the lowest proportion of CDS gene length to genome length. The strain DBM-5 comprised the highest GC content. Overall, the number of tRNAs in the non-coding RNA of the four strains was higher than that of the other RNA, with strain DX-3 having the highest number of tRNAs (Table 2).

The annotated gene ratios predicted by the genome sequencing of four strains through databases such as NR, COG, PFAM, GO, KEGG, CAZy, and CARD were presented in Figure S2. The NR database has the highest annotated ratio, with over 99.88% of genes successfully annotated. Strain DX-3 showed a higher gene annotation ratio in the COG, PFAM, GO, KEGG, CAZy, and CARD databases compared to the other three strains, especially in GO databases. Moreover, the gene annotation ratio was three times higher than other strains. 

Strains DBM-1, DX-3, and DX-4 have 713, 863, and 729 gene annotations in the GO database, respectively. All these strains annotate 33 functional classifications. In contrast, strain DBM-5 had a total of 2282 gene annotations, which cover 39 functional classifications (Figure S3). The genomes of these four strains exhibit the highest number of annotated genes in the biological process (BP) functional classification of the GO database, which was consistent. Cellular processes (GO: 0009987) and metabolic processes (GO: 0008152) were the central pathways in BP. The main pathway in molecular function (MF) was catalytic activity (GO: 0003824), while the main pathway in cellular component (CC) was membrane (GO: 0016020).

**Figure 4:**
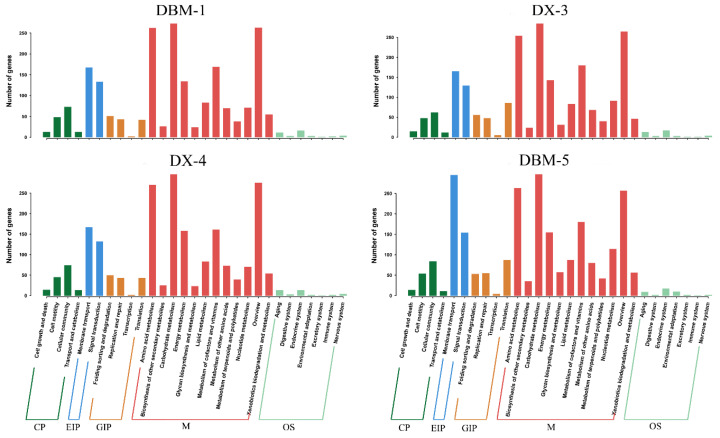
KEGG functional annotation of the genome of proteolytic bacteria.

Strains DBM-1, DX-3, DX-4, and DBM-5 have 1226, 1308, 1258, and 1580 genes annotated in the KEGG database, respectively (Figure 4). The number of genes annotated to the metabolism pathway was the highest, mainly enriched in carbohydrate metabolism and amino acid metabolism. The second highest was the environmental information processing pathway, mainly enriched in the membrane transport pathway. Notably, the genome of strain DBM-5 contained twice as many membrane transport genes compared to other strains. In the genetic information processing pathway, the number of genes involved in the transcription and translation pathways in the genomes of strains DX-3 and DBM-5 was significantly higher than in strains DBM-1 and DX-4.

Strains DBM-1, DX-3, DX-4, and DBM-5 have 74, 80, 79, and 100 genes annotated in the CAZy database, respectively (Figure S4). Among them, the largest number of genes were annotated to glycosyl transferases (GTs), followed by glycoside hydrolases (GHs). Strain DBM-5 exhibited a higher number of genes annotated to GTs, GHs, carbohydrate esterases (CEs), and auxiliary activities (AAs) compared to other strains. However, only polysaccharide lyases (PLs) were annotated in strains DX-3 and DX-4 genomes. The representative carbohydrate enzyme was pectate lyase (EC 4.2.2.2), which is involved in hydrolyzing the polysaccharides found in plant cell walls, suggested that strains DX-3 and DX-4 were also capable of breaking down plant materials [37].

The genomes of strains DBM-1, DX-3, DX-4, and DBM-5 predicted eight, six, eight, and nine secondary metabolite gene clusters, respectively [38] (Table 3). The main types of metabolites include terpene, lassopeptide, type III polyketide synthase (T3PKS), and non-ribosomal peptide synthase (NRPS), which participated in encoding surfactants, carotenoids, and non-ribosomal lipopeptides. It was observed that the predicted metabolites of the eight gene clusters in the genomes of strains DBM-1 and DX-4 were consistent; however, there were slight differences in similarity with known gene clusters. Additionally, all six predicted gene clusters in the genome of strain DX-3 were included in the gene clusters of strains DBM-1 and DX-4 but did not contain lanthiopeptide-class-i and phosphonate. Furthermore, the genome of strain DBM-5 was found to predict a completely different metabolic product gene cluster from other strains, with the primary metabolic product type being non-ribosomal peptide synthase (NRPS). 

We retrieved information on various types of proteases, including serine protease, pepsin, trypsin, cysteine protease, and metalloprotease from the Expasy database. Subsequently, we conducted a comparison and analysis of this information with the annotation results of the strain genome in KEGG ENZYME database. The results (Table 4) indicated that strains DBM-1, DX-3, DX-4, and DBM-5 harbored three, three, three, and four protease gene sequences, respectively. These sequences corresponded to serine protease, pepsin, and trypsin; however, no data regarding cysteine protease or metalloprotease were found in these strains. Notably, strain DBM-5 exhibited two distinct types of annotated trypsin within its genome.

## 4. Discussion

Cultivation of microbial technology is a traditional method for studying the interaction between hosts and symbiotic microorganisms, as well as for exploring functional genes. However, due to the large population of herbivorous insects [2], research on gut functional microorganisms mainly focuses on screening and identifying the microorganisms that hydrolyze lignin and cellulose [39,40]. While microbial proteases are important industrial enzymes, there have been few reports on the research of proteases derived from insect guts. Especially in Orthoptera, research on gut microorganisms has mostly focused on the structure, function, and relationship with the host life activities of the gut microbiota. This study utilized a selective culture medium to screen for proteolytic bacteria from the gut of *G. orientalis*, which was beneficial for exploring the functional microbial resources derived from the gut of Orthoptera. Four protease-producing microorganisms were preliminarily screened and identified in the gut of *G. orientalis*. Among these, *P. megaterium* was identified as an important enzyme-producing strain capable of producing various enzymes such as proteases, cellulases, oxidases, polysaccharide-degrading enzymes, extracellular agarases, etc. [41]. The probiotic properties of this bacterium play a positive role in maintaining gut health, and this study was the first to screen for the gut-derived *P. megaterium* that produces protease [42].

Identification and classification are two of the most important aspects of microbiology. The current phylogeny of prokaryotes is based on the 16S rRNA gene sequence (≥99%) [43]. However, the highly conserved sequence of the 16S rRNA gene limits the accuracy of species identification in prokaryotic diversity. Therefore, it is imperative to make improvements in this area to enhance the accuracy and specificity of species identification [44]. Raman spectroscopy, single genes, multigene, SNPs, and core-genome sequences [45] have emerged as powerful methods for microbial identification. However, whole-genome sequencing analysis [46] has been gradually applied to studying bacterial classification, evolution, and functional genes due to its superior ability to comprehend bacterial genomic information. Additionally, the assessment of inter-strain correlation based on whole-genome sequence ANI values (≥95%) and dDDH values (≥ 70%) has become a new gold standard for inter-species molecular identification [36]. This study preliminarily identified the taxonomic status of protease-producing strains using morphological, physiological and biochemical characteristics, as well as 16S rRNA gene sequencing. Furthermore, the species information of four strains was accurately identified based on whole-genome ANI and dDDH values [34,47]. Although the ANI and dDDH values of *P. aryabhattai* and *P. megaterium* initially classified under *Bacillus* exceeded the threshold; however, specific values can be used to determine differences between strains. To further clarify the functional information of the strains, whole-genome sequencing was utilized to comprehensively characterize the genetic basis of the strains, including genome composition, functional classification, and secondary metabolite synthesis gene clusters. Additionally, we investigated the protease gene information within the genomes of the strains and observed that strain DBM-5 possessed a greater variety of proteases compared to the other strains. This observation was consistent with the elevated protease activity identified during the screening of strain DBM-5. 

## 5. Conclusions

This study represents the first attempt to isolate protease-producing bacteria from the gut of *G. orientalis*. Four novel proteolytic bacteria were screened and identified as *P. aryahattai* DBM-1 and DX-4, *P. megaterium* DX-3, and *S. surfactantfaciens* DBM-5. Among them, the strain DBM-5 possesses abundant membrane transport genes and carbohydrate metabolism enzymes. Strains DX-3 and DX-4 not only demonstrate the ability to hydrolyze proteins but also exhibit proficiency in hydrolyzing plant materials.

## Figures and Tables

**Table 1:** The dDDH value of genome comparison between proteolytic bacteria and similar strains.

Strains	*Priestia aryabhattai* B8W22	*Priestia aryabhattai* UASWS 1812-B29	*Priestia megaterium* ATCC 14581	*Priestia megaterium* P-NA14
DBM-1	64.1	91.9	72.8	72.6
DX-3	64.2	73.4	74.7	90.2
DX-4	63.9	91.4	72.9	72.4
Strain	*Serratia surfactantfaciens* YD25	*Serratia ureilytica* T6	*Serratia marcescens* ATCC13880	*Serratia nematodiphila* DZ0503SBS1
DBM-5	96.3	25.5	56.5	56.9

**Table 2:** Sequencing results date statistics of proteolytic bacteria.

Genome Information	DBM-1	DX-3	DX-4	DBM-5
Genome size/bp	4,894,934	5,042,448	5,135,523	5,036,594
GC/%	37.79	38.04	37.66	59.72
CDS/ratio (%)	4963/83.2	5130/83.67	5222/82.17	4667/88.54
rRNA	12	23	7	12
tRNA	45	107	35	80
ncRNA	7	7	7	19

**Table 3:** Predicted biosynthesis clusters of proteolytic bacteria.

Secondary Metabolite Gene Clusters			Strains/Similarity			
Metabolite Type	Nucleotide Length/bp	Similar Gene Clusters/Type	DBM-1	DX-3	DX-4	DBM-5
terpene	20,819	surfactin/NRP:lipopeptide	+/13%	+/13%	+/13%	—
terpene	20,849	carotenoid/terpene	+/50%	+/50%	+/50%	—
NI-siderophore	16,576	schizokinen/other	+/100%	+/50%	+/62%	—
lassopeptide	23,919	paeninodin/RiPP	+/60%	+/60%	+/80%	—
T3PKS	41,086	—	+	+	+	—
terpene	21,869	—	+	+	+	—
phosphonate	17,423	—	+	—	+	—
lanthipeptide-class-i	16,669	paenicidin A/RiPP:lanthipeptide	+/28%	—	—	—
lanthipeptide-class-i	23,288	—	—	—	+	—
prodigiosin	35,021	prodigiosin/polyketide	—	—	—	+/100%
thiopeptide	26,440	o-antigen/saccharide	—	—	—	+/14%
opine-like-metallophore	22,098	yersinopine/other	—	—	—	+/100%
RRE-containing	20,279	synechobactin C9/C11/13/14/16/A/B/C (other)	—	—	—	+/9%
NRPS	47,380	viobactin/NPR	—	—	—	+/46%
NRPS	45,787	—	—	—	—	—
betalactone	25,668	—	—	—	—	—
NRP-metallophore	51,398	trichrysobactin/cyclic trichrysobactin/chrysobactin/dichrysobactin (NRP)	—	—	—	+/46%
NRPS butyrolactone	59,588	rhizomide A/B/C (NRP)	—	—	—	+/100%

Notes: The symbol “+” represents positive, and the symbol “—” represents negative.

**Table 4:** Identification of protease genes in bacterial genomes.

Proteases		DBM-1	DX-3	DX-4	DBM-5
Serine protease EC 3.4.21.53		ctg00001_001478	ctg00002_001384	ctg00001_002979	ctg00001_000175
Pepsin EC 3.4.23.3		ctg00001_001049	ctg00002_001814	ctg00001_002548	ctg00002_002628
Trypsin	EC 3.4.21.1	ctg00004_004107	ctg00006_003884	ctg00003_004439	ctg00002_00254
	EC 3.4.21.2				ctg00001_001383
Cysteine protease					
Metalloprotease					

## Data Availability

The assembled genomes were submitted to GenBank Database (accession number: GCA_037164005.1; GCA_037163955.1; GCA_037164135.1; GCA_037163965.1). The raw sequencing data was submitted to NCBI SRA database (accession number: PRJNA1063775; PRJNA1060919; PRJNA1063778; PRJNA1063541).

## Supplementary Materials

The following supporting information can be downloaded at: https://www.mdpi.com/article/10.3390/insects15080629/s1, Figure S1: Morphological observation of strain DBM-5 in protease producing screening medium with prolonged cultivation time. (a) Cultivate for 24 h; (b) Cultivate for 48 h; (c) Cultivate for 7 days; Figure S2: Morphological observation of strain DBM-5 in protease producing screening medium with prolonged cultivation time; Figure S3: GO functional annotation of the genome of proteolytic bacteria; Figure S4: CAZymes functional annotation of the genome of proteolytic bacteria; Table S1: Analysis of physiological and biochemical characteristics of proteolytic bacteria; Table S2: Analysis of physiological and biochemical characteristics of proteolytic bacteria.
