# Supplementary material for: Screening and Identification of Protease-Producing Microorganisms in the Gut of Gryllotalpa orientalis (Orthoptera: Gryllotalpidae)
Source: Insects. 2024 Aug 21;15(8):629. doi: 10.3390/insects15080629 (PMC11354742; doi:10.3390/insects15080629)
Supplement: Supplementary file 1 [file insects-15-00629-s001.zip › insects-3141971-supplementary.pdf]

Supplemental Material

Screening and identification of protease producing microorganisms in the gut of *Gryllotalpa orientalis* (Orthoptera: Gryllotalpidae)

Xiang Zheng [1, 2], Lu Zhao [1], Fangtong Wu [1], He Zhou [1] and Fuming Shi [2, *]

1 Laboratory of Enzyme Preparation, Hebei Research Institute of Microbiology Co., Ltd., Baoding 071051, China; 569186912@163.com (X.Z.); 15832297069@139.com (L.Z.); yayawu710@hotmail.com (F.W.); zhouhebio@163.com (H.Z.)

2 College of Life Science, Institute of Life Science and Green Development, Hebei University, Baoding 071002, China

* Correspondence: shif_m@126.com

Supplemental Table S1 Analysis of physiological and biochemical characteristics of proteolytic bacteria

Test items	DX-4	DBM-1	DX-3	Test items	DX-4	DBM-1	DX-3
Glycerol	+	+	w	Esculin	+	+	+
Erythritol	—	—	—	Salicin	+	+	+
D-arabinose	—	—	—	D-cellobiose	+	+	+
L-arabinose	+	+	+	D-maltose	+	+	+
D-ribose	+	+	+	D-lactose	+	+	w
D-xylose	+	+	+	D-melibiose	+	+	+
L-xylose	—	—	—	D-Sucrose	+	+	+
Ribitol	—	—	—	D-trehalose	+	+	+
β-methyl-D-xyloside	—	—	—	Inulin	—	—	—
D-galactose	+	+	w	D-melezitose	—	—	+
D-glucose	+	+	+	D-raffinose	+	+	+
D-fructose	+	+	+	Starch	+	+	+
D-mannose	—	—	—	Glycogen	+	+	+
L-sorbitol	—	—	—	Xylitol	—	—	—
L-rhamnose	+	—	—	D-gentiobiose	+	+	+
Dulcitol	—	—	—	D-Toulon sugar	+	+	w
Inositol	+	—	—	D-lyxose	—	—	—
Mannose	+	+	+	D-tagatose	—	—	—
Sorbitol	—	—	—	D-fucose	—	—	—
α-methyl-D-mannose	—	—	—	L-fucose	—	—	—
α-methyl-D-glucoside	+	—	—	D-arabitol	—	—	—
N-acetyl glucosamine	+	+	+	L-arabitol	—	—	—
Amygdalin	+	+	+	Potassium gluconate	—	—	—
Arbutin	+	+	+	2-keto-gluconate	—	—	—
				5-keto-gluconate	—	—	—

Notes: The symbol "+" represents positive, "w" represents a weak positive, and the symbol "—" represents negative.

Supplemental Table S2 Analysis of physiological and biochemical characteristics of proteolytic bacteria

Test items	DBM-5	Test items	DBM-5
β-galactosidase	+	Glucose produced acid	+
Arginine dihydrolase	—	Mannitol produced acid	+
Lysine decarboxylase	+	Inositol produced acid	+
Ornithine decarboxylase	+	Sorbitol produced acid	+
Citric acid utilization	+	Rhamnose produced acid	—
H_2S production	—	Sucrose produced acid	+
Urease	—	Melibiose produced acid	—
Tryptophan deaminase	—	Amygdalin produced acid	+
Indole prodution	—	Arabinose produced acid	—
VP experiment	+	oxidase	—
Gelatin liquefaction	+		

Notes: The symbol "+" represents positive, "w" represents a weak positive, and the symbol "—" represents negative.

(a) (b) (c)

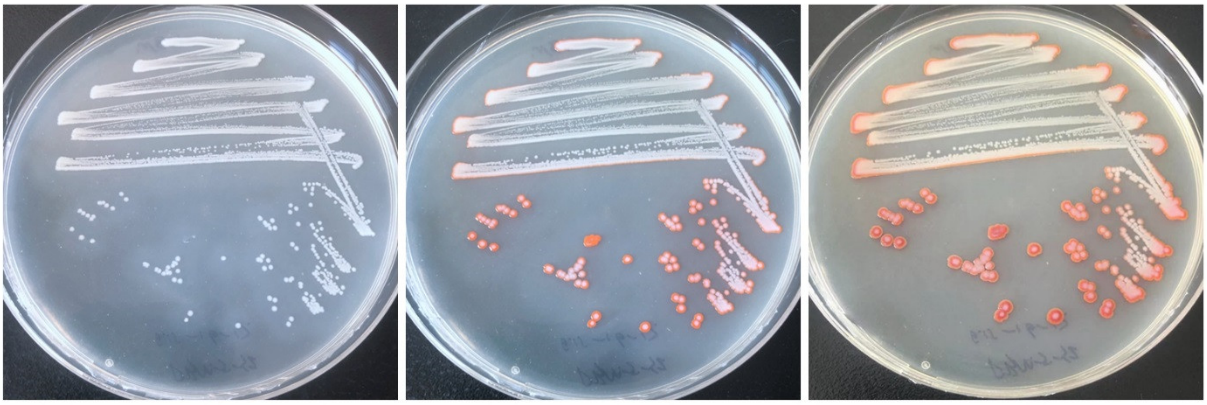

Supplemental Figure S1. Morphological observation of strain DBM-5 in protease producing screening medium with prolonged cultivation time. (a) Cultivate for 24 h; (b) Cultivate for 48 h; (c) Cultivate for 7 days.

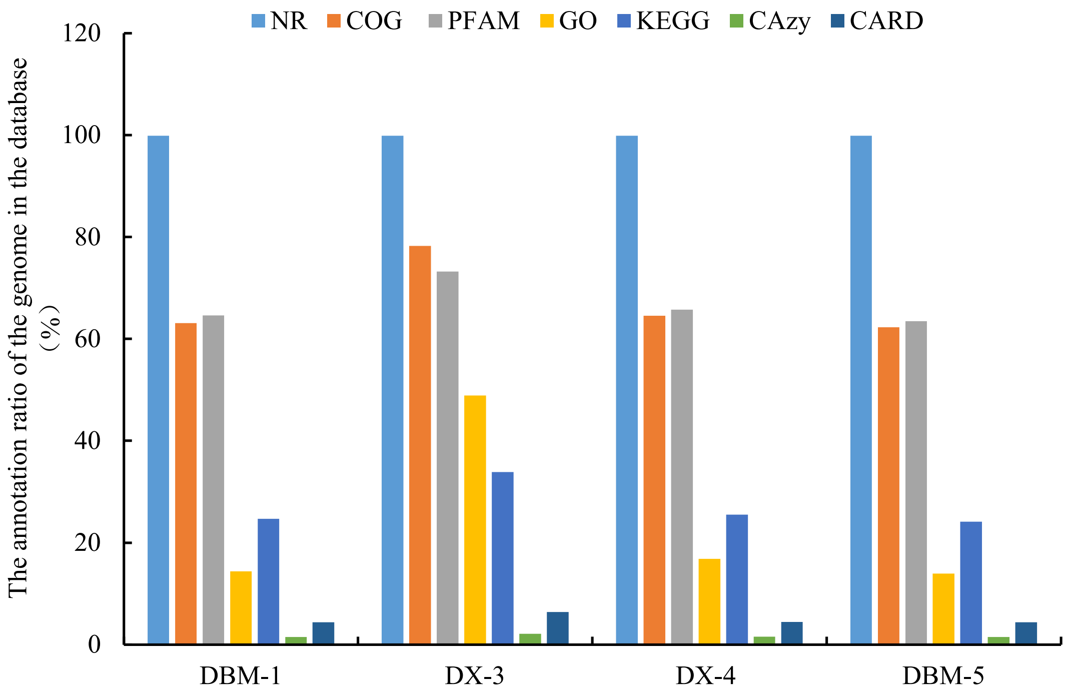

Supplemental Figure S2. Genome annotation of proteolytic bacteria

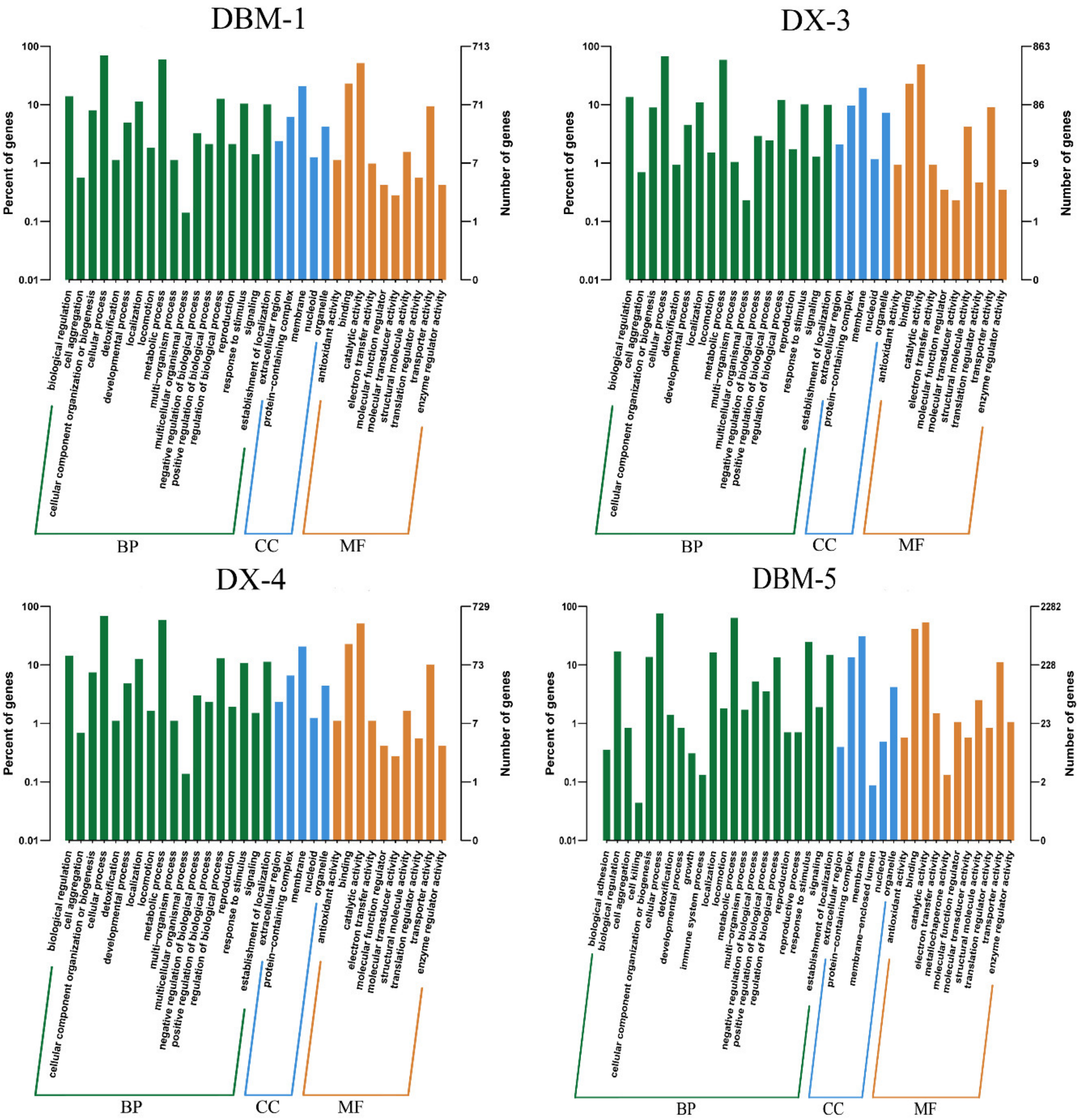

Supplemental Figure S3. GO functional annotation of the genome of proteolytic bacteria

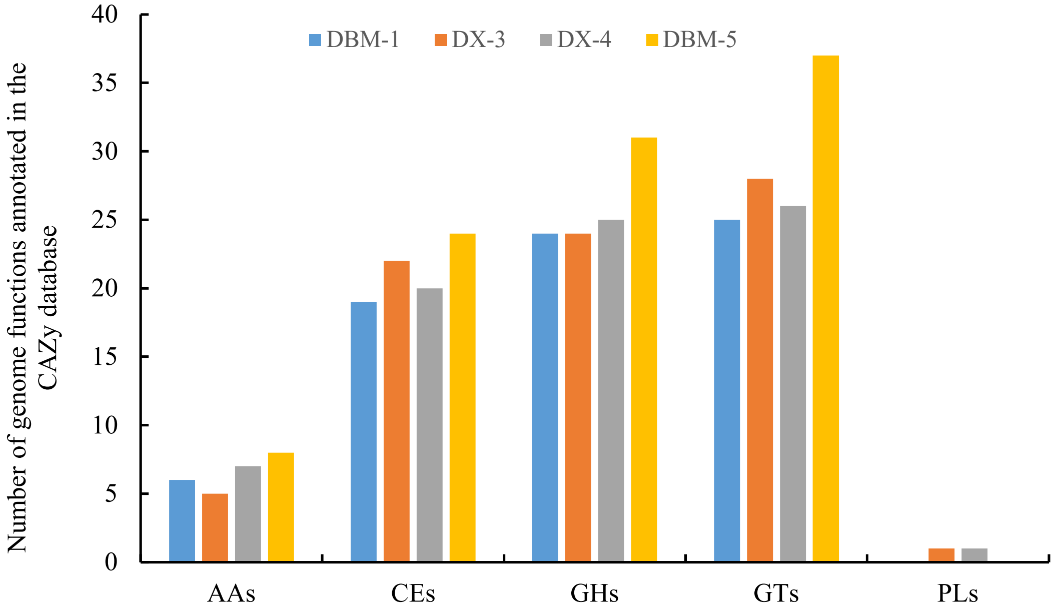

Supplemental Figure S4. CAZymes functional annotation of the genome of proteolytic bacteria
